# Association between *DSCAM* polymorphisms and non-syndromic Hirschsprung disease in Chinese population

**DOI:** 10.1186/s12881-018-0637-2

**Published:** 2018-07-13

**Authors:** Yong Wang, Qiuming He, Ruizhong Zhang, Wei Zhong, Deli Zhu, Yan Zhang, Huimin Xia

**Affiliations:** Department of Pediatric Surgery, Guangzhou Institute of Pediatrics, Guangzhou Women and Children’s Medical Center, Guangzhou Medical University, 9 Jinsui Road, Guangzhou, 510623 Guangdong China

**Keywords:** Hirschsprung disease, *DSCAM*, Association, Genetics

## Abstract

**Background:**

Hirschsprung disease (HSCR, aganglionic megacolon) is the most frequent genetic cause of congenital intestinal obstruction. *DSCAM* was identified as associated to HSCR with Down Syndrome (DS-HSCR) in European population,but failed to replicate in the non-syndromic HSCR patients. We aim to further investigate the relationship of *DSCAM* with non-sydromic HSCR in a South Chinese cohort, the largest case-control study so far.

**Method:**

We analyzed 1394 HSCR patients and 973 healthy controls. Two polymorphisms (rs2837770 A > G, rs8134673 A > G) on *DSCAM* were genotyped using Sequenom Massarray platform.

**Results:**

Both SNPs were confirmed as associated with non-syndromic HSCR in the South Chinese population (*P* = 1.69E-03, OR = 1.29 for SNP rs2837770 and *P* = 3.00E-03, OR = 1.27 for SNP rs8134637). Of note, we demonstrated the associated SNPs were more likely to affect a subgroup of patients with short-segment aganglionosis (S-HSCR) (*P* = 3.06E-03,OR = 1.21 for SNP rs2837770 and *P* = 3.33E-03,OR = 1.21 for SNP rs8134637).

**Conclusion:**

There is an association between *DSCAM* polymorphisms and non-syndromic HSCR in South Chinese population.

**Electronic supplementary material:**

The online version of this article (10.1186/s12881-018-0637-2) contains supplementary material, which is available to authorized users.

## Background

Hirschsprung disease (HSCR) is the most common, complex genetic disorder of the enteric nervous system (ENS) with an incidence of one in 5000 live births [1, 2]. It is characterized by the absence of enteric ganglia in the myenteric and submucosal plexuses along a variable length of the hindgut [3]. HSCR can occur in combination with other conditions, such as Waardenburg syndrome, Mowat-Wilson syndrome; or Downs syndrome which are described as syndromic. HSCR occur without other conditions were referred to as non-syndromic [2, 4]. Clinically, according to the length of the aganglionic segment, HSCR can be classified as short-segment HSCR (S-HSCR, 80% of cases) and long-segment HSCR (L-HSCR, 20% of cases) [5]. In few L-HSCR cases, the entire colon and even the whole bowel are involved and can be further classified into Total Colonic aganglionosis (TCA) and total intestine aganglionosis (TIA) (together less than 5% of cases). L-HSCR patients with longer aganglionic segments have been recognized as presenting poorer life quality and when compared to S-HSCR patients [6]. However, the etiology of the disease and its subclinical manifestations was still uncertain.

More than 15 loci have been identified as contributing to Hirschsprung disease, [7]. Most of them were involved in Enteric Neural Crest Cell (ENCC) proliferation, maintenance of Enteric nervous system (ENS) progenitors and neuronal or glial differentiation and hence only indirectly influence migration [7]. They were roughly classified into four major groups: *RET* and its ligands; *EDNRB* and *EDN3* and *ECE1* in the same pathway; the NRG homologs (*NRG1* and *NRG3);*and the *SEMA* signaling pathway (*SEMA3C and SEMA3D)* [2, 4]. Syndromes associated with HSCR are usually considered as monogenic diseases; Waardenburg syndrome type 4 (Waardenburg-Shah syndrome) was considered as 100% coincident with HSCR. Down syndrome (trisomy 21) is the most common chromosome abnormality associated with HSCR which occurs in 2-10% of all individuals with HSCR [8]. In contrast, approximately 3% of individuals with Down syndrome have HSCR [9].

Jannot et al. [10] revealed two common variants (rs2837770 and rs8134673) in *DSCAM* as associated with 26 Caucasian HSCR patients accompanied with Down syndrome. *DSCAM* is expressed at a high level in the developing nervous systems in human, mouse and drosophila, implying a crucial role for *DSCAM* in the development of the nervous system [11]. However, they failed to replicate the association using 220 non-syndromic HSCR trios and public data awaiting for further replication.

Hence, in order to identify the association of *DSCAM* with non-syndromic HSCR, we conducted a replication study on the two SNPs in *DSCAM* using 1394 sporadic HSCR cases and 973 controls in South Chinese population. Further experiment was still required to further digest its functional mechanism to both central nervous system (CNS) and ENS.

## Methods

### Study subjects

To investigate the association between the chosen genetic polymorphisms and the risk of HSCR, we totally included 1394 sporadic patients recruited from 2000 to 2015 who have received treatments from the Guangzhou Women and Children’s Medical Center. All the cases were diagnosed with HSCR by examination of biopsy specimen for the absence of the enteric ganglia. The detailed clinical information was summarized in Additional file 1 Table S1. Regarding to the severe status, HSCR patients was usually classified into S-HSCR, L-HSCR and TCA. After revisiting the clinical records, we divided the 1394 HSCR cases into three subgroups. The patients with/without Down Syndrome were further examined. There were five patients diagnosed with HSCR accompanied with Down syndrome, four were classified as S-HSCR and one of them considered as L-HSCR. During the same period, a group of 973 healthy, unrelated subjects visiting the same medical center for routine health check were randomly selected as a control group with matched gender and age. The study was approved by the institutional review board of Guangzhou Women and Children’s Medical Center, Guangzhou Medical University, (No.2016042036). The written informed consents were provided by guardians of all subjects.

### Polymorphism analysis

Two previously examined SNPs (rs2837770 A > G, rs8134673 A > G) in *DSCAM* were chosen for replication in this study MassARRAY iPLEX Gold system (Sequenom) was selected for genotyping on all the samples and we used PLINK1.9 (genotype test of 3 × 2 contingency tables, Cochran–Armitage trend test, test of dominant and recessive models) to compare the minor allele frequency in patients and controls [12]. Linkage disequilibrium patterns and values were obtained using HaploView [13].

### Haplotype association analysis

The two SNPs were phased using the E-M algorithm implanted in PLINK. The association was performed using logistic regression on two most likely haplotypes composed by the two SNPs.

### Immunohistochemistry staining

DSCAM expression pattern was assessed in a patient by comparing the aganglionic and ganglionic segments, together with non-HSCR colon tissue from patient undergoing colostomy through immunostaining. Colon samples from the HSCR patients and patient for colostomy in this study were obtained at the time of the surgery. Immunohistochemical staining for DSCAM was performed using 4 μm deparaffinized sections. The slides were prepared following standard procedures and stained with an anti-DSCAM primary antibody (Atlas Antibodies, HPA019324, 1:1000) and secondary antibody (anti-rabbit (DAKO, K4003)).

## Results

### Association of SNPs in *DSCAM* with HSCR susceptability

The genotype distributions for the two SNPs (rs2837770 and rs8134673) did not violate hardy weinberg equilibrium (HWE) (*P* = 0.4746 for rs2837770, *P* = 0.3559 for rs8134673) in the subjects. The genotype frequencies of the SNPs and the association to disease are summarized in Table 1. For SNP rs2837770, risk allele G was significantly associated with increased HSCR risk (OR = 1.16,95% CI = 1.03~ 1.31, *P* = 1.25E-02). For SNP rs8134673,consistent with SNP rs2837770,risk allele G elevated the risk of HSCR (OR = 1.16, 95% CI = 1.03~ 1.31, *P* = 1.69E-02). To better understand the effective patterns for the two associated SNPs, we specified the patients and controls into genotypic, dominant and recessive patterns (Table 1). We observed the main effect to disease for both SNP were based on a recessive model. The associations following the recessive model exhibited 1.3-folds (rs2837770 and rs8134673) risk with disease for both SNPs. We also performed haplotypic association based on the two SNPs, two alleles“A-A” composed as the protective haplotype for HSCR showed the most significant association with disease (*P* = 7.45E-03)(Table 2).

**Table 1:** Replication results on two selected SNPs in *DSCAM* in South Chinese population using 1470 cases and 943 controls

SNP	Chr	BP	Gene	feature	left_gene	right_gene	A1/A2	F_A	F_U	P	OR	95% CI
*P_hwe = 0.05*							G/A					
rs2837770	21	40,956,222	*DSCAM*	intron[NM_001389.3]	*PCP4*	*BACE2*	ADD	1169/1619	860/1026	1.25E-02	1.16	(1.03~ 1.31)
							GENO	251/667/476	181/498/264	7.08E-03	–	–
							DOM	918/476	679/264	4.68E-01	1.08	(0.88~ 1.34)
							REC	251/1143	181/762	1.74E-03	1.33	(1.11~ 1.58)
rs8134673	21	40,970,181	*DSCAM*	intron[NM_001389.3]	*PCP4*	*BACE2*	ADD	1184/1604	867/1019	1.69E-02	1.16	(1.03~ 1.31)
							GENO	255/674/465	184/499/260	1.21E-02	–	–
							DOM	929/465	683/260	4.59E-01	1.08	(0.88~ 1.34)
							REC	255/1139	184/759	3.05E-03	1.32	(1.10~ 1.58)

*SNP* Single Nucleotide Polymorphism, *Chr* Chromosome, *BP* Base pair of where the SNP is located, *Gene* The gene where the SNP located to; Feature: the feature of the variants in the gene;left/right_gene: the upstream/ downstream gene where the variant located; A1/A2 indicates the risk allele and protective allele to disease; F_A/F_U indicates risk allele numbers of the SNP in cases or controls. The *P* value indicates the significance based on different association tests. The calculation of odds ratio (OR) and the 95% confidence of intervals (CI) is also based on the risk allele of each SNP

**Table 2:** The haplotype association testing of two SNPs in DSCAM in South Chinese population using 1473 cases and 943 controls

NSNP	Chr	BP1	BP2	SNP1	SNP2	HAPLOTYPE	F_A	F_U	OR	P
2	21	40,662,426	40,676,385	rs2837770	rs8134673	AA	0.42	0.46	0.85	7.45E-03
2	21	40,662,426	40,676,385	rs2837770	rs8134673	GG	0.58	0.54	1.14	0.027

*NSNP* Number of Single Nucleotide Polymorphism, *Chr* Chromosome, *BP* Base pair of where the SNP is located; *HAPLOTYPE* the haplotype composed by two *SNPs* F_A/F_U indicates risk allele frequency of the haplotype in cases or controls. The calculation of odds ratio (OR) was based on the haplotype of patients. The P value indicates the significance based on different association tests

We examined the relationship between the two identified SNPs with HSCR using our results (Guangzhou,GZ). Consistent with the public data including East Asians (EA) and Caucasians (CEU) downloaded from 1000 Genome database, high correlation was observed between the two SNPs with r^2^ equal to 0.95 in our replication. This piece of data reflect the two SNPs were derived from one associated signals to disease (Additional file 2 Figure S1).

### Clinical stratification of SNPs in *DSCAM* with HSCR

We further stratified the subclinical features of the HSCR patients by the length of the aganglionic segment and syndromic symptoms. Due to the limited Down syndromic HSCR sample size, we failed to elaborate and replicate the association of the two SNPs with this specific symptom accompanied by HSCR. In terms of association to different aganglionic status, the two SNPs were observed consistent patterns as associated with different type of aganglionosis through subphenotype-control analysis, including S-HSCR versus controls, L-HSCR versus controls and TCA versus controls respectively (Table 3). It seems the two SNPs are more likely to affect S-HSCR patients rather than L-HSCR and TCA patients. We further examined the association of SNPs with other subclinical stratification including enteritis before and after operation, gender, by case-only testing, however, no obvious significant association was observed (data not shown Additional file 3 Table S2).

**Table 3:** The association results of two SNPs in DSCAM to different subclinical features classified by aganglionois length including short-length (S-HSCR), long-length (L-HSCR) and TCA

Length of Aganglionosis	CHR	SNP	BP	A1/A2	F_A	F_U	*P*	OR	CI 0.95
SHSCR	21	rs2837770	40,662,426	G/A	0.59	0.54	*3.06E-03*	1.21	(1.07~ 1.38)
	21	rs8134673	40,676,385	G/A	0.59	0.54	*3.33E-03*	1.21	(1.07~ 1.38)
LHSCR	21	rs2837770	40,662,426	G/A	0.56	0.54	*0.63*	1.05	(0.86~ 1.27)
	21	rs8134673	40,676,385	G/A	0.54	0.54	*0.91*	1.01	(0.84~ 1.22)
TCA	21	rs2837770	40,662,426	G/A	0.56	0.54	*0.66*	1.07	(0.78~ 1.48)
	21	rs8134673	40,676,385	G/A	0.57	0.54	*0.2*	1.12	(0.81~ 1.55)

*CHR* Chromosome, *SNP* Single Nucleotide Polymorphism, *BP* Base pair of where the SNP is located. A1/A2 indicates the risk allele and protective allele to disease; F_A/F_U indicates risk allele frequency of the SNP in cases or controls. The P value indicates the significance based on allelic association tests. The calculation of odds ratio (OR) is also based on the risk allele of each SNP

### Functional characterization of DSCAM in colon

The expression of DSCAM was observed in both myenteric plexus and mucosa reflecting a widely expression of DSCAM in colon. We observed an obvious descending across normal tissue to aganglionic tissue (Additional file 4 Figure S2). We also pursued the systemic biological protein network centered by *DSCAM* gene using GeneMANIA [14], showing a strong interaction with *PAK1,* which was reported as playing key roles to regulate cell motility and morphology (Additional file 5 Figure S3). However, we failed to affirm the potential regulating roles of the two associated SNPs with the expression of DSCAM in current study.

## Discussion

The exact cause of HSCR is unknown. In an attempt to confirm the association of *DSCAM* with HSCR, we collected 1394 patients and 943 controls in South Chinese population, and successfully replicated two SNPs in *DSCAM* as associated with non-syndromic HSCR, especially in patients affected with a short anglia segment. We also observed a distinct lower expression in aganglionic segment compared with ganglionic segment and normal controls. Of course, the underlying mechanism to HSCR is still unclear which requires for further examination.

*DSCAM* has been repeatedly reported as a crucial player in neural development in vertebrates. It mediates homophilic attraction in neuronal layer-targeting [15]. Recently *DSCAM* has been identified as another receptor for netrin-1 [16]. It also works as a receptor for netrin in mediating axon guidance [17]. In addition to the contribution to DS-HSCR patients demonstrated by Jannot et al. [10], our results point to *DSCAM* as a susceptibility locus to non-syndromic HSCR patients. We also observed that the association of the two replicated SNPs mainly come from the effect on S-HSCR patients, which is similar to the associated common variants in *RET* mainly affecting the sporadic S-HSCR patients [18]. They also found rare mutations in *RET* were more likely to affect the severe cases including L-HSCR and TCA cases. At this stage, the predisposition of *DSCAM* to S-HSCR may due to the limited samples for L-HSCR patients and TCA patients, we don’t have enough power to detect the hidden association signals. It is also possible the common variants only play roles with S-HSCR patients with moderate effect, and there may exist rare variants which serve as casual mutations to the severe cases of the disease. Further study is required to validate the association in an independent cohort or directly search the potential functional rare variants by target region sequencing on *DSCAM*.

The lower expression of *DSCAM* was observed in the aganglionic segment of HSCR in current study. However, there still exist a gap how the associated SNPs may regulate the expression of DSCAM. We failed to identify the potential roles for the two SNPs through RegulomDB annotations [19]. Further experiments were required to further digest how *DSCAM* may affect the disease status of HSCR.

## Conclusions

In conclusion, our study provides further evidence that two SNPs rs2837770 and rs8134673 in *DSCAM* contributed to the risk of non-syndromic HSCR. Lower expression of DSCAM was observed in the aganglionic segment of HSCR patients revealed it may play key roles in enteric ganglia formation. Our study proposed a link that may help bridge the gap between genetic susceptibility and disease etiology, functional studies are required to strengthen our findings.

## Additional files

Additional file 1: **Table S1.** The subclinical information collected for the subjects in this study. (PDF 68 kb)

Additional file 2: **Figure S1.** The LD (r^2^) patterns of two SNPs in *DSCAM* in Guangzhou replication (Guangzhou), East Asian (EA) and Caucasian (CEU) populations from 1000G data. (http://grch37.ensembl.org/Homo_sapiens/Tools/VcftoPed?db=core). (PDF 193 kb)

Additional file 3: **Table S2.** The Case-only subclinical association analysis. (PDF 158 kb)

Additional file 4: **Figure S2.** The expression of DSCAM in the intestinal mucosa of (A) a non-HSCR subject. (B) dilated (ganglionic) segment and (C) narrow (aganglionic) segment of a HSCR patient. (PDF 138 kb)

Additional file 5: **Figure S3.** DSCAM shows strong connection with PAK1 analyzed by GeneMania. (PDF 138 kb)

## Abbreviations

CEU: Caucasians
DSCAM: Down syndrome cell adhesion molecule
DS-HSCR: HSCR with Down Syndrome
EA: East Asians
ENCC: Enteric neural crest cell
ENS: Enteric nervous system
GZ: Guangzhou
HSCR: Hirschsprung disease
HWE: Hardy weinberg equilibrium
L-HSCR: Long-segment HSCR
S-HSCR: Short-segment aganglionosis
TCA: Total Colonic aganglionosis
TIA: Total intestine aganglionosis
