# Supplementary material for: Association between DSCAM polymorphisms and non-syndromic Hirschsprung disease in Chinese population
Source: BMC Med Genet. 2018 Jul 13;19:116. doi: 10.1186/s12881-018-0637-2 (PMC6045829; doi:10.1186/s12881-018-0637-2)
Supplement: Supplementary file 1 — Table S1. The subclinical information collected for the subjects in this study. (PDF 68 kb) [file 12881_2018_637_MOESM1_ESM.pdf]

Supplementary Table 1. The subclinical information collected for the subjects in this study.

HCSR subphenotype	Cases (n=1470)		%	Controls (n=1473)	%
Subjects					
Age range（Months）	8.37±20.50			18.61±19.75	
≤2	725		49.32%	458	31.09%
>2	745		50.68%	1015	68.91%
Clinical manifestation	DS	Non-DS			
S-HCSR	4	1029	70.27%		
L-HCSR	1	293	20.00%		
TCA	0	82	5.58%		
Total intestine	0	3	0.20%		
Enteritis_before_operation	261		17.76%		
Enteritis_after_operation	249		16.94%		
