# Supplementary material for: Association between DSCAM polymorphisms and non-syndromic Hirschsprung disease in Chinese population
Source: BMC Med Genet. 2018 Jul 13;19:116. doi: 10.1186/s12881-018-0637-2 (PMC6045829; doi:10.1186/s12881-018-0637-2)

Supplementary Figure 1. The LD (r^2) patterns of two SNPs in *DSCAM* in Guangzhou replication (Guangzhou), East Asian (EA) and Caucasian (CEU) populations from 1000G data. (http://grch37.ensembl.org/Homo_sapiens/Tools/VcftoPed?db=core)

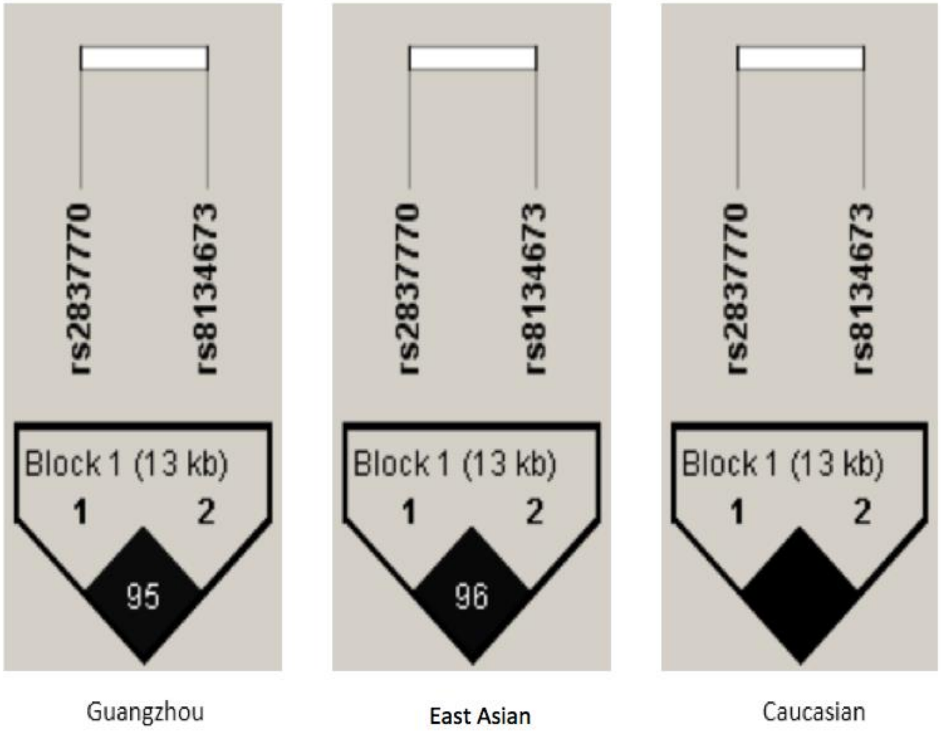

Supplement: Supplementary file 2 — Figure S1. The LD (r2) patterns of two SNPs in DSCAM in Guangzhou replication (Guangzhou), East Asian (EA) and Caucasian (CEU) populations from 1000G data. (http://grch37.ensembl.org/Homo_sapiens/Tools/VcftoPed?db=core). (PDF 193 kb) [file 12881_2018_637_MOESM2_ESM.pdf]
