# Supplementary material for: Association between DSCAM polymorphisms and non-syndromic Hirschsprung disease in Chinese population
Source: BMC Med Genet. 2018 Jul 13;19:116. doi: 10.1186/s12881-018-0637-2 (PMC6045829; doi:10.1186/s12881-018-0637-2)
Supplement: Supplementary file 3 — Table S2. The Case-only subclinical association analysis. (PDF 158 kb) [file 12881_2018_637_MOESM3_ESM.pdf]

Supplementary Table 2. The Case-only subclinical association analysis.

CHR	SNP	BP	A1/A2	F_A	F_U	P	OR(CI 0.95)
Enteritis before operation							
21	rs2837770	40662426	G/A	0.57	0.59	0.37	0.90(0.73~1.13)
21	rs8134673	40676385	G/A	0.56	0.58	0.39	0.91(0.73~1.13)
Enteritis after operation							
21	rs2837770	40662426	G/A	0.53	0.59	0.04	0.80(0.64~0.99)
21	rs8134673	40676385	G/A	0.54	0.58	0.13	0.84(0.68~1.05)
Gender							
21	rs2837770	40662426	G/A	0.58	0.58	0.88	0.99(0.81~1.20)
21	rs8134673	40676385	G/A	0.57	0.57	0.74	0.97(0.79~1.18)

CHR：chromosome; SNP: single nucleotide polymorphism; BP: base pair; A1/A2: minor/major allele of the polymorphism; F_A/F_U: Minor allele frequency of the affected/unaffected subjects; OR: Odds Ratio; CI: Confidence of interval.
