# Supplementary material for: Association between DSCAM polymorphisms and non-syndromic Hirschsprung disease in Chinese population
Source: BMC Med Genet. 2018 Jul 13;19:116. doi: 10.1186/s12881-018-0637-2 (PMC6045829; doi:10.1186/s12881-018-0637-2)

**Supplementary Figure 2**. The expression of DSCAM in the intestinal mucosa of (A) a non-HSCR subject. (B) dilated (ganglionic) segment and (C) narrow (aganglionic) segment of a HSCR patient.

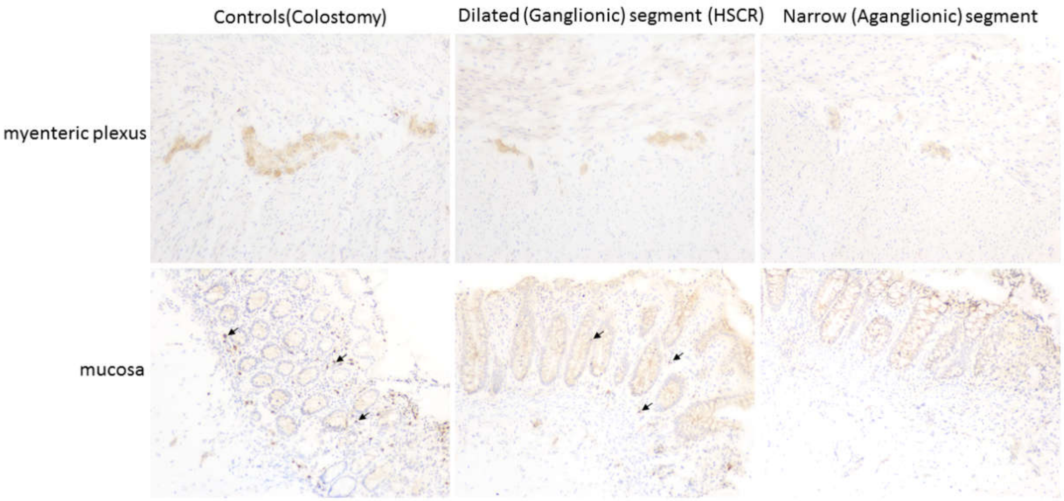

Supplement: Supplementary file 4 — Figure S2. The expression of DSCAM in the intestinal mucosa of (A) a non-HSCR subject. (B) dilated (ganglionic) segment and (C) narrow (aganglionic) segment of a HSCR patient. (PDF 138 kb) [file 12881_2018_637_MOESM4_ESM.pdf]
