# Supplementary figures and images for: Association between DSCAM polymorphisms and non-syndromic Hirschsprung disease in Chinese population
Source: BMC Med Genet. 2018 Jul 13;19:116. doi: 10.1186/s12881-018-0637-2 (PMC6045829; doi:10.1186/s12881-018-0637-2)

**Supplementary Figure 3.** DSCAM shows strong connection with PAK1 analyzed by GeneMania.

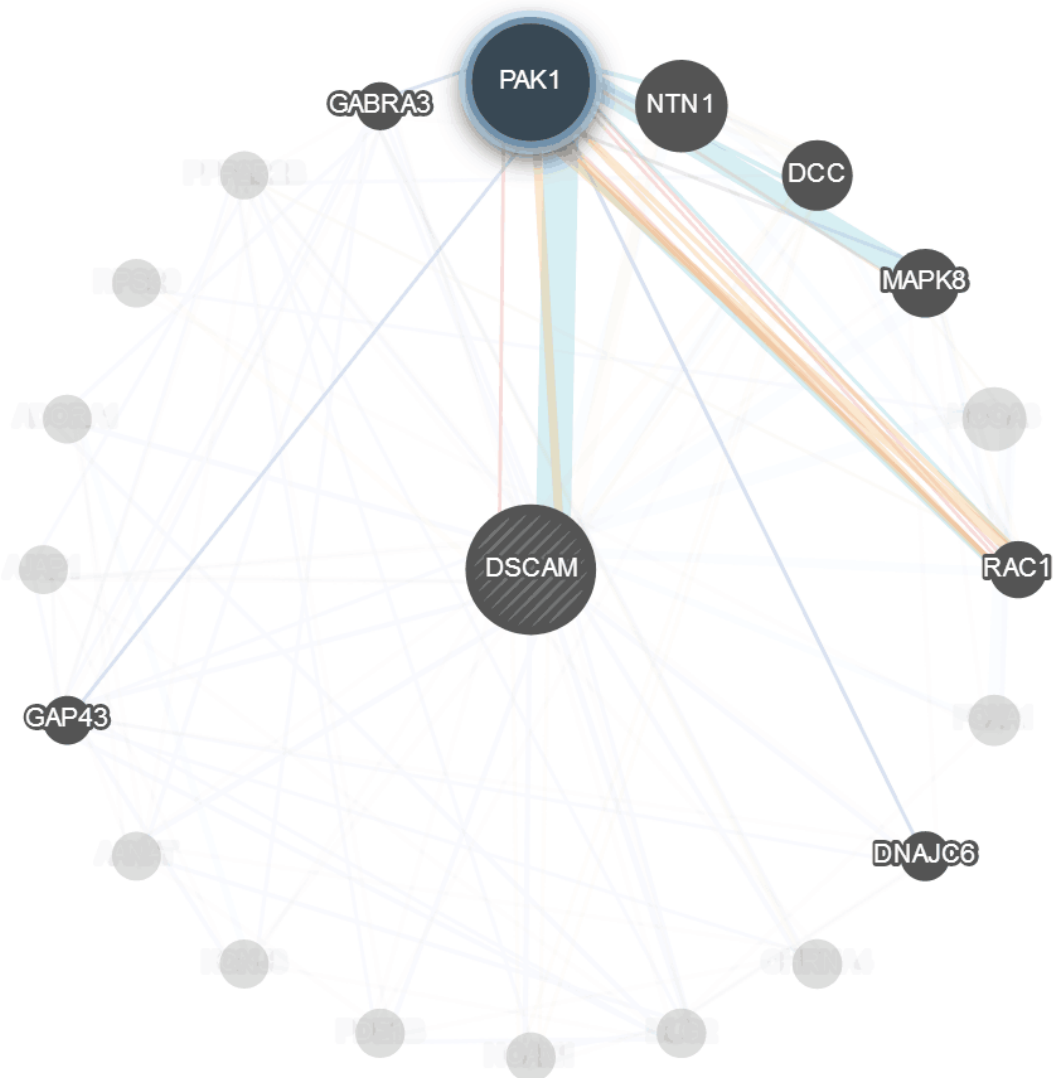

Supplement: Supplementary file 5 — Figure S3. DSCAM shows strong connection with PAK1 analyzed by GeneMania. (PDF 138 kb) [file 12881_2018_637_MOESM5_ESM.pdf]
